# Resonantly Enhanced Difference-Frequency Generation in the Core X-ray Absorption of Molecules

**DOI:** 10.1021/acs.jpca.1c06950

**Published:** 2021-12-15

**Authors:** Carles Serrat

**Affiliations:** Department of Physics, Polytechnic University of Catalonia, Colom 11, 08222 Terrassa (Barcelona), Spain

## Abstract

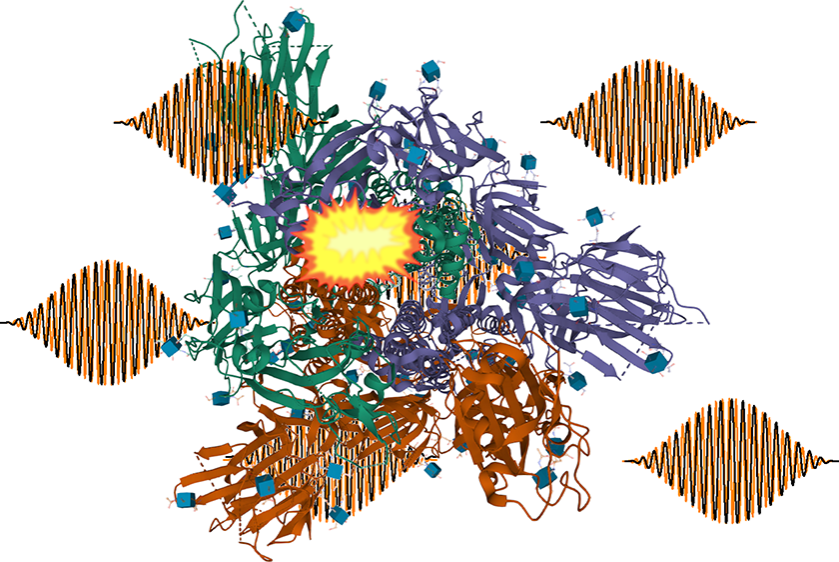

We use real-time time-dependent density functional theory simulations to numerically demonstrate that resonantly enhanced difference-frequency generation (re-DFG) involving intense ultrashort coherent X-ray pulses can selectively excite core states of atoms in molecules. As a model case, we evaluate the spectral selectivity of re-DFG excitation of the oxygen K-edge by illumination of a single gas-phase water molecule with two-color X-ray pulses of different photon energies and durations. The re-DFG excitation is further probed by a small delayed pulse with central photon energy resonant with the oxygen K-edge peak absorption line. Based on these results, we anticipate that highly selective excitation by re-DFG X-ray nonlinear processes might be achieved in more complex molecular systems and bulk materials by using highly penetrating two-color hard X-ray pulses, with extensive applications.

## Introduction

Selective photodissociation of molecules by resonant excitation near a chosen core ionization edge by means of nonlinear interactions in the X-ray range is becoming conceivable due to recent advances in the development of intense ultrashort X-ray coherent pulse sources, such as synchrotron, free-electron lasers (FELs), and in high-harmonic generation.^1−10^

Several nonlinear interactions in the X-ray range are accordingly being investigated.^11^ In a previous work,^12^ we showed how the phase-sensitivity cancellation of the anti-Stokes component previously described in two- and three-level systems in the infrared and optical regions can be extended to the X-ray absorption near edge structure (XANES) and extended X-ray absorption fine structure (EXAFS)^6^ of chemical species by highly localized four-wave-mixing (FWM) nonlinear processes. Femtosecond transient FWM grating spectroscopy with ultrafast X-rays has recently been demonstrated,^13,14^ showing experimentally how the large penetration depth of X-rays allows probing the bulk properties of materials, addressing core excited states, and creating excitation gratings with unprecedented nanoscale spatial resolution. Among the X-ray wave-mixing processes, difference-frequency generation (DFG) of optical and UV radiation using two-color (Ω_1_, Ω_2_) X-ray laser pulses, where ℏΩ_*i*_ is the central photon energy of the pulses, has been studied theoretically.^15,16^ We note here that atomic units and hence ℏ = 1 are used in the rest of the paper.

Here we address atomic core resonantly enhanced difference-frequency generation (re-DFG) from X-ray pulses, considering the oxygen K-edge in a single gas-phase water molecule as a model system. We numerically show how the illumination of a water molecule with two-color (3Ω, 4Ω) femtosecond X-ray pulses produces a difference-frequency component Ω that is enhanced at the core resonance Ω = ω_0_, with ω_0_ being in this case the oxygen K-edge in the gas-phase water molecule, and evaluate the degree of excitation selectivity as a function of the duration of the two-color pulses.

## Numerical Simulations

**Figure 1:**
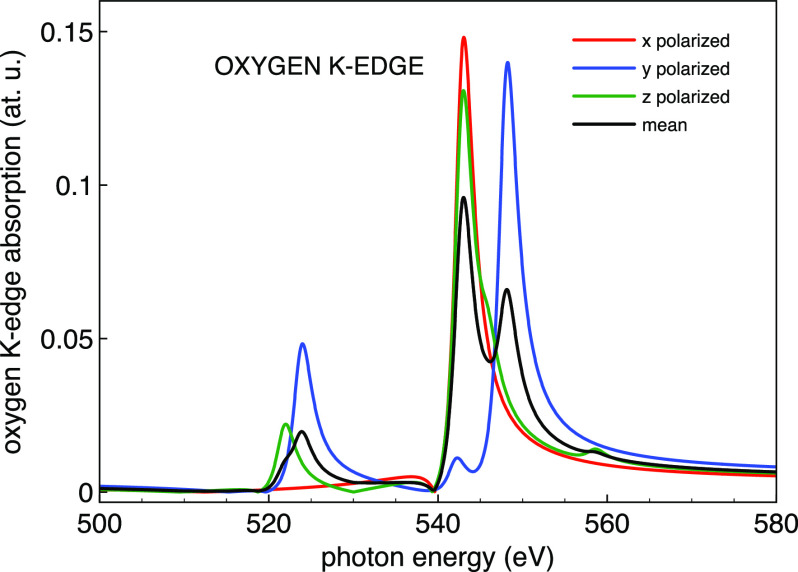
Oxygen K-edge linear absorption lines obtained from the imaginary part of the Fourier transform of the time-dependent dipole moments, after the system has been excited with a small electric field kick of 10^–4^ au ≈ 51 mV/nm at *t* > 0. The peaks have been broadened to account for core relaxation effects by damping the dipole time signal by e^–*t*/*t*_d_^, with *t*_d_ = 20 au ≈ 480 as. The absorption obtained with different linear field polarizations together with the mean in the three directions is shown.

The first step is to calculate the absorption spectrum of the molecule around the target absorption edge. Figure 1 shows the computed water linear spectrum considering different field polarizations, together with the absorption spectrum obtained by adding the different polarization contributions. The simulations have been performed using the NWChem real-time TDDFT module.^17^ A delta-function electric field perturbation applied at *t* = d*t*, with d*t* = 0.045 au, excites all electronic modes of the system as
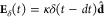
1where κ is the kick strength and **d̂** is the unit polarization vector in Cartesian coordinates. The complex molecular polarizability components α̃_*i*_(ω) in the *x*, *y*, and *z* directions are obtained from the Fourier transform of the dipole moments μ_*i*_(*t*), which are computed separately, divided by the Fourier transform of the corresponding electric field component *E*_δ_*i*__(*t*) as
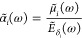
2with *i* = *x*, *y*, *z*. The absorption spectrum *S*_1_(ω) is then calculated with the mean of the imaginary part of the polarizability components as
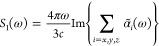
3

It is intricate to determine the convenient choice of exchange-correlation functionals and basis sets to describe the absolute positions, number of spectral peaks, and relative strengths of a particular system. An extensive study of the gas-phase water molecule was performed in ref (18), where the B3LYP functional together with Sapporo basis sets gave the best overall agreement with experimental results. In Figure 2 of ref (18), a comparison between the theoretical and experimental results for the absorption spectrum of the gas-phase water molecule around the oxygen K-edge can be examined. After testing several exchange-correlation functionals and basis sets, in the present work, we have chosen the Gaussian basis set aug-cc-pVTZ for the nuclear geometry optimization in the *y*–*z* plane together with the exchange-correlation functional PBE0, and instead the relatively small 6-31G basis set with PBE0 for the real-time TDDFT. The absorption spectrum around the oxygen K-edge that we obtain coincides reasonably well with the experiments.^18,19^ The calculated oxygen K-edge main absorption peak is neat at 543 eV, which differs by 7 eV from the experimental value, and the near-edge features agree in number and relative strengths although they span a larger frequency domain. We hence resolve that the theory level that we use is sufficiently accurate and most computationally efficient for the real-time TDDFT description of the fundamental physics of the re-DFG process that we address, which is based on the resonance at the main oxygen K-edge spectral peak.

In order to examine the re-DFG nonlinear effect, the water molecule is illuminated with two-color (3Ω, 4Ω) laser pulses and the resulting DFG component is evaluated by varying Ω. The two-color pulses are linearly polarized in the *x*, *y*, and *z* directions, with all polarizations in phase with each other, so that the resulting polarization is not aligned with any of the molecular axis. The two-color field components in each direction have the form
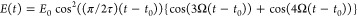
4where *E*_0_ is the peak amplitude of the two-color pulse components, τ gives the duration of the pulses, which in the present simulations are 1.67, 2.56, and 3.43 fs (fwhm), 3Ω and 4Ω are the respective central angular frequencies, and *t*_0_ centers the pulses in the temporal grid. An important matter with respect to the potential applications of the re-DFG effect that we discuss is the balance between the peak intensity needed for the nonlinear effect to occur and the maximum peak intensity for extended molecular damage. A laser peak intensity of 10^12^ W/cm^2^ has been chosen in the present simulations as an example to be about the order of magnitude for harmless doses in different tissues and materials as reported in ref (20) and as will be further commented below. Additionally, in Figure 4, a study of the variation of the re-DFG signal as a function of the laser pulse peak intensities and their photon energies is shown.

**Figure 2:**
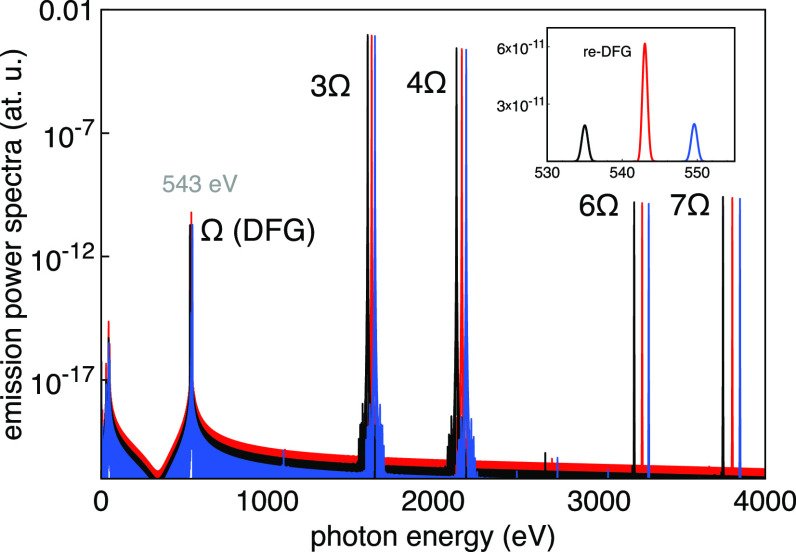
Power spectra *D*(ω) obtained by Fourier transform of the acceleration of the time-dependent dipole resulting from the interaction of the two-color (3Ω, 4Ω) pulses with the water molecule, for three different values of Ω. The inset is a zoom of the spectra near the oxygen K-edge showing the enhancement of the DFG signal at resonance (Ω = ω_0_ = 543 eV).

**Figure 3:**
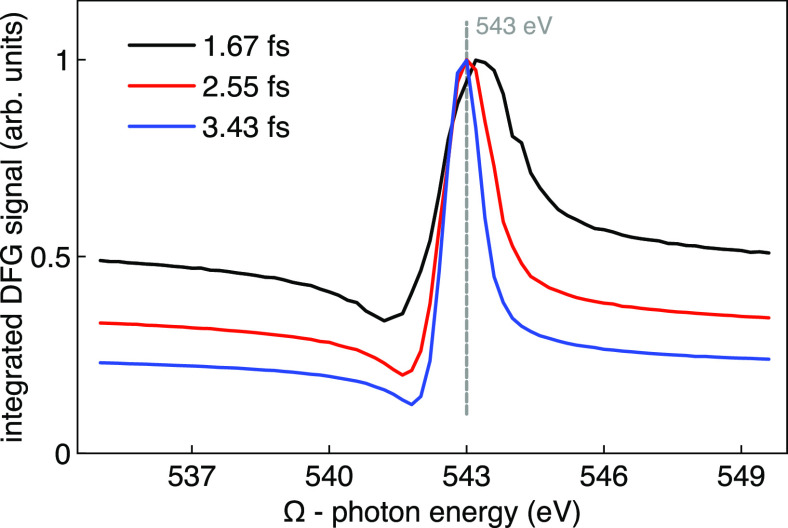
Integrated DFG spectral signal around the oxygen K-edge as a function of the incident photon energy (Ω) of the two-color (3Ω, 4Ω) pulses.

The power spectra shown in Figure 2 have been obtained by computing the Fourier transform of the acceleration of the expectation value of the time-dependent dipole *d*(*t*) = μ̈(*t*) resulting from the interaction of the two-color pulses with the system as^21^
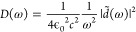
5A Hann-type time window of the form  is applied before the Fourier transform, where *t*_T_ is the total time of the simulation. Figure 2 shows the spectral signal *D*(ω) obtained for three different central photon energy values of the incident two-color pulses that produce a DFG component very close to the calculated oxygen K-edge. The inset shows how the signal peaks at the oxygen K-edge, which is the demonstration that the DFG signal is enhanced by the core oxygen K-edge resonance (ω_0_ = 543 eV). Other frequency mixing effects can also be seen in Figure 2 at larger spectral values, corresponding to the second harmonic of the 3Ω field (6Ω) and the sum-frequency generation 3Ω + 4Ω signal (7Ω). The central photon energy of the input two-color pulses (3Ω, 4Ω) has been scanned by varying Ω to give DGF signals from about 535 to 550 eV, and the resulting spectrally integrated DFG signal is plotted in Figure 3 for three different durations of the two-color input pulses, as indicated. The integration of the power spectra has been considered in the region around the oxygen K-edge, spanning the frequency range between 500 and 600 eV. Here we can clearly see the peak of the integrated DFG signal at the oxygen K-edge (543 eV), which, as can be expected, it is narrower as the duration of the incident pulses is increased. The integrated DFG values in Figure 3 have been normalized for clarity. It is worth noting that, in order to optimize the computational requirements of our rt-TDDFT simulations, the durations of the pulses that we have considered are remarkably short (1.67, 2.56, and 3.43 fs), and therefore, much narrower peaks in Figure 3 can be expected by considering longer incident pulses, which would consequently result in a more selective re-DFG effect. Markedly, they can be most regularly produced in X-ray FEL experiments.

After the two-color (3Ω, 4Ω) laser pulse interacts with the water molecule, the system remains core excited due to re-DFG when Ω = ω_0_ = 543 eV, since no relaxation processes are considered in the simulations. We observe how the differences between the excited and ground state charge densities^18^ follow complicated dynamics around the oxygen atom, with a periodicity that corresponds to the calculated oxygen K-edge, therefore demonstrating 1s core excitation (not shown).

**Figure 4:**
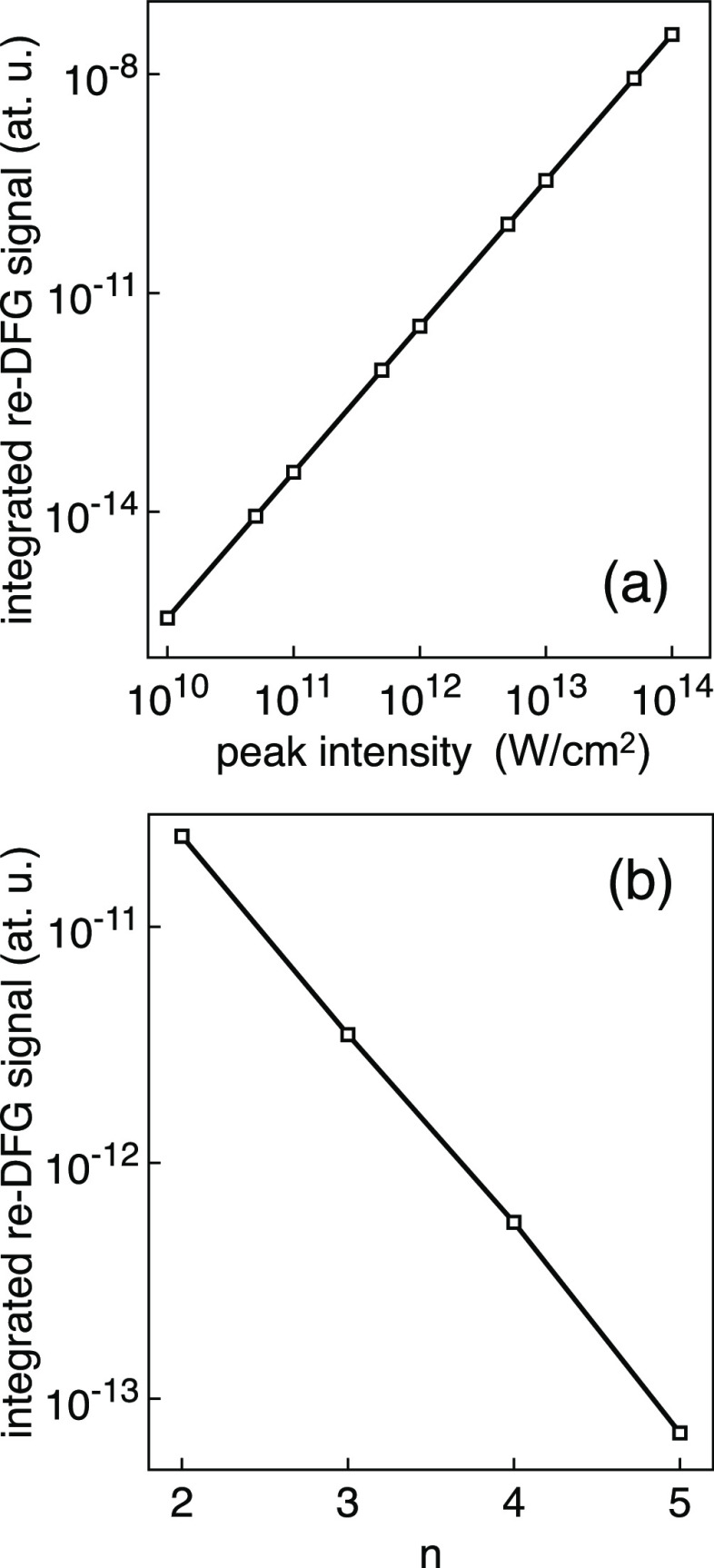
(a) Log–log plot showing the quadratic dependence of the re-DFG integrated signal on the peak intensity of the laser pulses, in the case of 3.43 fs two-color (3ω_0_, 4ω_0_) pulses (ω_0_ = 543 eV). (b) Dependence of the re-DFG integrated signal on the photon energy input pulses as (*n*ω_0_, (*n* + 1)ω_0_), in the case of 3.43 fs pulses with 10^12^ W/cm^2^ peak intensity.

Figure 4 shows the dependence of the re-DFG integrated signal on the peak intensity of the laser pulses, in the case of two-color (3ω_0_, 4ω_0_) pulses (Figure 4a), together with the variation of the re-DFG integrated signal as a function of the photon energy input two-color pulses as (*n*ω_0_, (*n* + 1)ω_0_), with 10^12^ W/cm^2^ peak intensity (Figure 4b), and for 3.43 fs pulses in both cases. In the log–log plot of Figure 4a, we can observe how the intensity of the re-DFG signal increases as the square of the intensity of the input laser pulse, as is expected from a second-order nonlinear polarization. Figure 4b gives the computed decay of the re-DFG integrated signal as the photon energy of the two-color pulses is considered further away from the oxygen K-edge resonance, with a dependence on *n* of approximately (*n*^2^ + *n*)^−4^, as can be determined from the second order nonlinear susceptibility, although the precise values will count on the decay rate of the dipole.^22^

**Figure 5:**
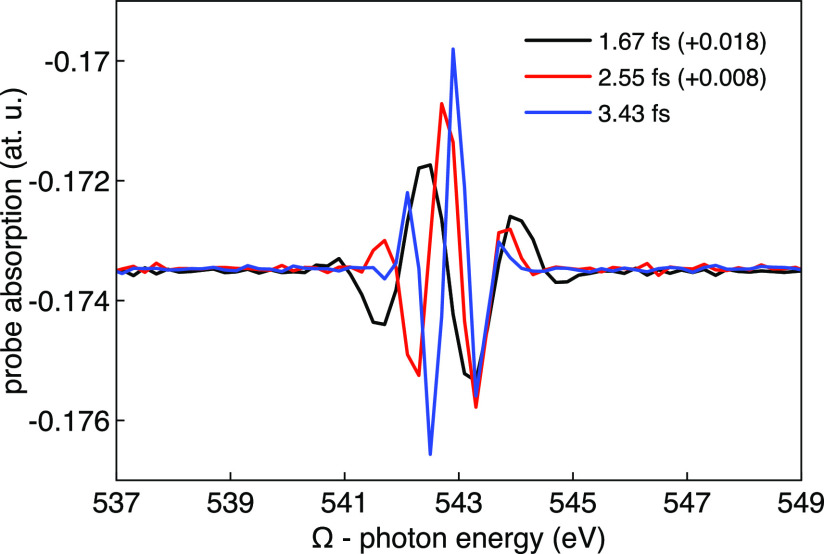
Probe absorption (*S*_2_) as a function of the incident photon energy (Ω) of the two-color (3Ω, 4Ω) pulses. The curves have been slightly shifted vertically as indicated for a better visualization of the oscillation width.

In order to evaluate in an all-laser-field approach the degree of excitation of the oxygen after the interaction with the two-color (3Ω, 4Ω) pulse, we add a cos^2^ envelope probe pulse of 0.8 fs (fwhm) duration and 10^4^ W/cm^2^ peak intensity, with the central photon energy of the calculated oxygen K-edge (ω_0_ = 543 eV). The probe is delayed 240 as from the end of the input two-color pulse. The absorption spectrum *S*_p_(ω) of the probe pulse in the medium is computed in the frame of transient absorption signals, as described in ref (23)
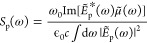
6where *Ẽ*_p_(ω) is the complex spectrum of the probe pulse and μ̃(ω) is the Fourier transform of the time-dependent dipole, considered in the complete pump–probe time window of the calculation. The total probe absorption signal is then obtained by integration of *S*_p_(ω)
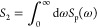
7Negative (positive) values of *S*_p_(ω) or *S*_2_ correspond to absorption (emission) of light. The absorption cross section *S*_2_ multiplied by the number density of atoms results in the absorption/gain coefficient. The computed probe absorption around the oxygen K-edge as a function of the central photon energy of the input two-color pulses (3Ω, 4Ω) is shown in Figure 5. We observe clear oscillations of the absorption rate around the oxygen K-edge, which provide the evidence that the atom is excited about 543 eV. Also clear in Figure 5 is the spectral width of the excitation, which is set by the duration of the input pulses, as commented above. The period of the oscillations of the probe absorption in Figure 5 corresponds to the Fourier limited bandwidth of the input pulses, which is roughly 1.6 eV for the 1.67 fs pulses, 1.1 eV for the 2.55 fs pulses, and 0.8 eV for the 3.43 fs pulses, and it is a consequence of the periodic convolution between the probe field and the nonlinearly DFG generated time-dependent dipole moment, which modulates their time delay as Ω is varied and therefore also the probe absorption rate.^23^

## Discussion and Conclusions

In conclusion, we have numerically shown that two-color (3Ω, 4Ω) re-DFG can be implemented using synchronized femtosecond X-ray pulses for the selective excitation of atomic core states, considering a single gas-phase water molecule as a model system. The duration of the two-color pulses determines the spectral selectivity that can be achieved by the nonlinear re-DFG effect. We have shown how the re-DFG integrated signal increases as the laser peak intensities increase and decreases as the photon energy of the two-color pulses is increased. It is deserving to comment that rt-TDDFT has shown a fail in describing resonant excitations at relatively high intensities (10^11^–10^12^ W/cm^2^).^24^ This is certainly not the case of the generated re-DFG signal in our study, which is several orders of magnitude less intense than the input pulses, always in the linear regime, and therefore, the time-dependent dipole resulting from its resonant core interaction with the atom is accurately described.^18,25^ It is also worth noting that our simulations build upon the dipole approximation, so that some corrections due to non-dipole effects are expected at the high photon energies that we consider, to which extent would demand further theoretical and experimental studies.^26^

The results that we have described might be extended to other molecules, essentially considering that the interaction of coherent X-ray FEL pulses is locally produced at the site specific atoms rather than the whole molecule. Since DFG is a second-order nonlinear effect, some symmetry-breaking is expected and would not be produced, for instance, in rare gas atoms. The effect in solids and other more complex molecules needs to be investigated separately and using the appropriate level of theory in each case. In this regard, it is well-known that resonant excitation near a core atomic ionization edge in a molecule can follow a rapid redistribution of charge beginning in less than a femtoseconds via Auger processes, so that multiple charged molecular ions can be formed and the molecule generally will become unstable and dissociate.^27^ From this perspective, considering that a spectral fingerprint of the particular state of a molecule can generally be obtained by its XANES/EXAFS linear absorption spectra at the different atomic absorption edges, the selectivity of the re-DFG process that we outline could further be optimized using a combination of several synchronized two-color (Ω_1_, Ω_2_) pulses, which can produce re-DFG signals resonant with several XANES/EXAFS absorption lines simultaneously, resulting in additional selective local excitation of the molecule.

Beyond the fundamental and applied interest of the nonlinear re-DFG X-ray matter interaction process that we have presented in a single gas-phase water molecule, our motivation also anticipates the potential of extending it to higher photon energies following the rapid advances in FEL sources, such as considering two-color hard X-rays to core excite higher atomic number atoms by re-DFG. The essential part of this hard X-ray approach is that the two-color (Ω_1_, Ω_2_) pulses can be highly penetrating in bulk, while the core resonant re-DFG signals Ω_2_ – Ω_1_ are not. Therefore, by virtue of the results from the water model that have been shown, we may consider re-DFG to be achieved in the atomic edges of, e.g., biomolecular zinc complexes^28^ (Zn K-edge ∼10 keV), using two-color (Ω_1_, Ω_2_) pulses of highly penetrating Ω_1_ and Ω_2_ hard X-ray central photon energies. Consistently, it was recently reported that exposure to laser-produced hard X-ray pulses with relatively high peak intensities does not lead to increased harm to mammalian cells exposed in vitro compared with the harm induced from exposure to hard X-rays from conventional medical sources, concluding that the use of high-power laser facilities for medical imaging is justified.^20^ In this regard, the optimal two-color pulse photon energies, peak intensities, and durations to attain a harmless dose in different tissues and materials using hard X-ray re-DFG need to be addressed by additional molecular dynamics studies together with experimental measurements. We can hence envisage extensive potential applications of the nonlinear X-ray re-DFG effect that we describe, which include, in general, the local and selective manipulation of atoms and molecules in bulk matter, and, in particular, in medicine, it might be significant for local and selective cancellation of the active center of biomolecules.^28−30^
